# Prevalence of overweight and obesity in preschool children (2–5 year-olds) in Birjand, Iran

**DOI:** 10.1186/1756-0500-5-529

**Published:** 2012-09-25

**Authors:** Taheri Fatemeh, Hassanzadeh-Taheri Mohammad-Mehdi, Kazemi Toba, Nazari Afsaneh, Gholamreza Sharifzadeh

**Affiliations:** 1 Pediatrics Department, Birjand University of Medical Sciences, Ghaffary St, 9717853577, Birjand, Iran; 2 Anatomical Sciences, Anatomy Department, Birjand University of Medical Sciences, Ghaffary St, 9717853577, Birjand, Iran; 3 Birjand Atherosclerosis and Coronary Artery Research Center, Cardiology, Birjand University of Medical Science, South khorassan, Pasdaran Street, Birjand, Iran; 4 Birjand University of Medical Science, Birjand, Iran; 5 Birjand University of Medical Sciences (BUMS), Ghaffary St, 9717853577, Birjand, Iran

**Keywords:** Obesity, Overweight, Preschool children, BMI (Body Mass Index)

## Abstract

**Background:**

Childhood overweight and obesity have increased progressively in developing countries and nowadays they are considered as a global epidemic.

The aim of the present study was to determine prevalence of overweight and obesity in 2–5 year-old children in kindergartens of Birjand in 2008.

**Findings:**

This cross- sectional and descriptive- analytical study was conducted on 500 children, who were 2–5 years-old, in kindergartens of Birjand selected through census. It was found that prevalence of overweight was 10.6% (11.7% in females and 9.6% in males) and obesity 7.6% (6.3% in females and 9.6% in males). Prevalence of overweight and obesity were statistically significant regarding birth weight, mother's occupation and father's level of education.

**Conclusion:**

Prevalence of overweight and obesity in pre-schoolchildren is more than that of 7–18 year-old group in Birjand, but it is less than the result of similar studies in Tehran and most studies in other countries. Further studies are recommended to identify risk factors in obese children. Periodic studies are necessary to compare the changes in prevalence of obesity in children in future.

## Background

The incidence of chronic diseases is spreading much more rapidly in developing countries than in developed ones [1]. A marked increase in the prevalence of overweight and obesity has been observed in the last few decades, both in adults and in children worldwide [2]. Childhood overweight and obesity have increased progressively in developing countries but nowadays they are considered as a global epidemic [3].

Interestingly, in many communities which still suffer from malnutrition, simultaneous growing prevalence of overweight and obesity are reported [4].

Analysis of 144 studies in different countries, during 2010, showed that 43 millions of preschool children were overweight or obese, while 35 millions of them belonged to developing countries, and 92 millions were at the risk of becoming overweight. Furthermore, overweight and obesity in preschool children is growing rapidly, so that it reached from 4.2% in 1990 to 6.7% in 2010. It is predicted that it will reach to 9.1% in 2020 which will cover 60 million children. In Asia, prevalence of overweight and obesity in preschool children was 4.9% in 2010 which covered 18 million children [5].

Changes in the life style, urbanization, mal-nutrition, and use of high calorie fast foods and also decreased physical activity, due to watching TV and playing sedentary games, are significant causes of children's obesity [3].

Childhood obesity is associated with serious health problems like increasing morbidity, so that out patient visits, hospitalization and treatment in obese children is more than non-obese ones [6].

In addition, childhood obesity is associated with premature complications in the youth, i.e. increasing of obesity risk and its related diseases. They are the major health challenges in the world and considered as a leading cause of death [5]. On the other hand, childhood and adolescence obesity are important precursors of adult obesity [7].

Diagnosis and control, as well as prevention and treatment of obesity, should start early- in life, and the first thing to do is to identify different aspects of the problem. Then, you need to get more information about the prevalence of childhood obesity in different areas for effective intervention.

The present study was designed to determine the prevalence of overweight and obesity in 2–5 year-old children in Birjand and their relationship with some variables such as; birth- weight, duration of breastfeeding, rank of birth, number of children in the family, level of parents' education, occupations of parents and the length time of watching TV during a day.

## Methods

**Figure 1:**
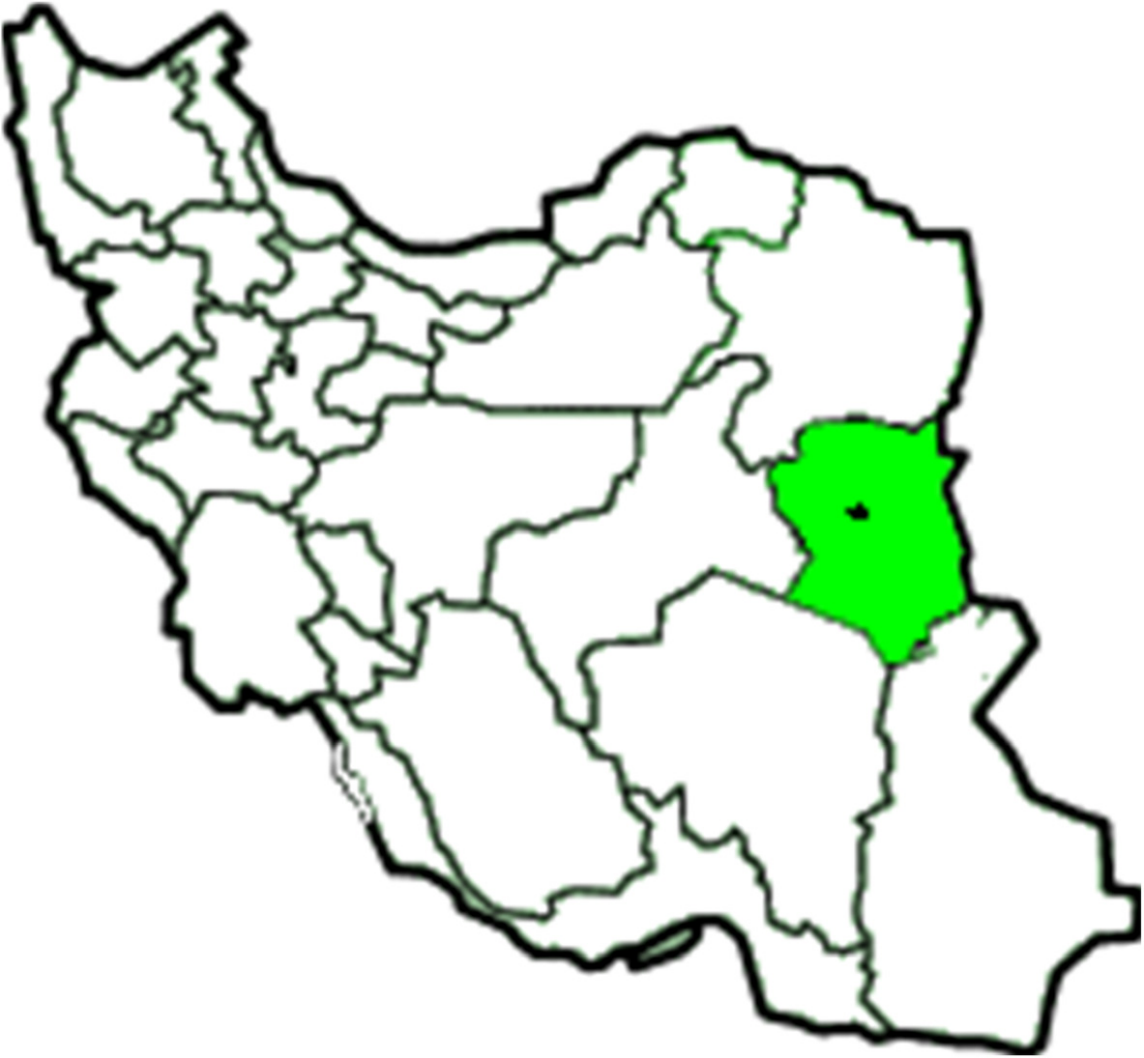
Map of Iran showing geographic regions and the location of Birjand.

This descriptive- analytical cross-sectional study was done on 2–5 years old in the urban area of Birjand in 2008. Birjand,the capital of South-Khorasan province, is a city,having a population of about 180,000, is located in the east of Iran and is one of the most deprived areas of the country (Figure 1).

The subjects included 500(239 girls and 261 boys) from all four socio-economic areas of the city,who were selected through census, after obtaining permission from welfare and education organizations.

Obtaining data was based on clinical examination of height and weight The height of every subject was measured by means of a tape with the margin of error of 0.5 centimeter. The weight of every child was measured using a German scale (Seka) with the margin error of 50 grams,which was calibrated by means of a control scale every day. Moreover,all children were weighed with light suits on .Besides, all measurements were done by a trained medical student employing the same tools.

To gather data, a researcher designed questionnaire whose reliability had been approved by five academic members in pediatrics department was used. The questionnaire had questions regarding demographic queries, number of children in the family, birth rank, parents' education, parents' jobs, and period of watching TV which were answered through interviewing each one's mother. Questions regarding birth weight and breast milk feeding period of every child were filled out by referring to respective health ICs of the children in their files in their kindergarten office.

Children who had acute diseases (e.g. diarrhea, acute infection on the reference of the researchers), children whose parents disagreed the study, and children who took corticosteroids were excluded from the study.

Body Mass Index (BMI) as weight (Kg) ratio to squared height (m^2^) was calculated for each child. To determine overweight and obesity, BMI percentile and CDC were used. BMI between 85 and 95 percentiles, (for age and sex), was accounted as overweight and greater than 95 is defined as obese. Each age- group was compared with the median age in CDC charts. Thereafter, the obtained data was coded, processed and analyzed by means of SPSS software (version 11.5) and differences less than 0.05 (P < 0.05) were considered significant.

In order to compare the prevalence of relative frequency of overweight and obesity in different genders and age-groups and their relationship with other variables, chi-square test was used.

Besides, agreement to carry out the present research was obtained from Birjand University of Medical Sciences Ethics Committee.

## Findings

In this study, 500 children (239 girls and 261 boys) were studied. According to BMI criteria, 10.6% of children were overweight and 7.6% obese. Prevalence of overweight and obesity were 11.7% and 6.3% in girls and 9.6% and 8.8% in boys respectively .Thus the differences were statistically non-significant (Table 1). Prevalence of overweight and obesity accounting for age and gender is shown in Table 2.

**Table 1:** Frequency of overweight and obesity and other variables in 2–5 year-old children in Birjand kindergartens

**Statistical Index Variables**		**N (%)**	**Total N (%)**	**Obese N (%)**	**Overweight N (%)**	**P value**
age	2.5 ± 0.5	108	108 (21/6)	5 (4.6)	8 (7.4)	
	3.5 ± 0.5	149	149 (29/8)	13 (8.7)	17 (11.4)	0.46
	4.5 ± 0.5	243	243 (48/6)	20 (8.2)	28 (11.5)	
**Birthweight**	< 2500 gr	46	46 (9/2)	1 (2.2)	3 (6.5)	0.002
	≥ 2500 gr	454	454 (90/8)	37 (8.1)	50 (11)	
**Mother’s job**	House wife	202	202 (40/4)	21 (10.4)	14 (6.9)	0.02
	Employed	298	298 (59/6)	17 (5.7)	39 (13.1)	
**Father’s job**	Free	150	150 (30)	13 (8.7)	18 (12)	
	Employment	280	280 (56)\	19 (6.8)	27 (9.6)	0.85
	Other	70	70 (14)	6 (8.6)	8 (11.4)	
**Mother’s level of education**	< Diploma	41	41 (8/2)	7 (17.1)	4 (9.8)	0.07
	Diploma	162	162 (32/4)	14 (8.6)	13 (8)	
	> Diploma	297	297 (59/4)	17 (5.7)	36 (12.1)	
**Father’s level of education**	< Diploma	35	35 (7)	5 (14.3)	6 (17.1)	0.01
	Diploma	176	176 (35/2)	17 (9.7)	10 (5.70)	
	> Diploma	289	289 (57/8)	16 (5.5)	37 (12.8)	
**Birth rank**	First to third	481	481 (96/2)	37 (7.7)	52 (10.8)	0.54
	Forth & more	19	19 (3/8)	1 (5.3)	1 (5.3)	
**Number of children**	≤ 3	475	475 (95)	31 (7.6)	52 (10.9)	0.59
	> 3	25	25 (5)	2 (8)	1 (4)	
**Breast feeding**	≤ 20 Month	151	151 (30/2)	11 (7.3)	11 (7.3)	0.27
	> 20 Month	349	349 (69/8)	27 (7.7)	42 (12)	
**Daily Watching TV**	≥ 2 hours daily	304	304 (60/8)	20 (6.6)	26 (8.6)	0.08
	> 2 hours daily	196	196 (39/2)	18 (9.2)	27 (13.8)	

**Table 2:** Absolute and relative frequency of overweight and obesity in 2–5 year-old children in Birjand based on age and sex

**Age (year)**	**Total N (%)**	**Sex N (%)**	**Overweight N (%)**	**Obese N (%)**	**Normal or Low birth weight N (%)**
2.5 ± 0.5	46 (42/6)	Female	3 (6.5)	2 (4.3)	41 (89.1)
	62 (57/4)	Male	5 (8.1)	3 (4.8)	54 (87.1)
3.5 ± 0.5	78 (52/4)	Female	6 (7.7)	7 (9)	65 (83.3)
	71 (47/6)	Male	11 (15.4)	6 (8.5)	54 (76.1)
4.5 ± 0.5	**90 (46/2)**	Female	19 (16.5)	6 (5.2)	90 (78.3)
	**105 (53/8)**	Male	9 (7/1)	14 (10.9)	105 (82)
Total	**500 (100)**	Female + Male	53 (10.6)	38 (7.6)	409 (81.8)

As it is shown in Table 1, comparing the prevalence rates between overweight and obesity with age on one hand, and gender, on the other, revealed no significant difference.

Prevalence of obesity with respect to birth weight (< 2500 g and ≥ 2500 g) was 2.2% and 8.1%, respectively and that of overweight was 6.5% and 11%; therefore, the differences were statistically significant.

Prevalence of obesity regarding mother's occupation (housewife and employed) were 10.4% and 5.7% and that of overweight was 9.6% and 13.1% respectively;thus, the differences were statistically significant.

Prevalence of obesity regarding father's level of education (lower than diploma, diploma, and higher than diploma) were 14.3%, 9.7% and 5.5% respectively and for overweight it was 17.1%, 5.7% and 12.8% respectively;therefore, the differences were statistically significant. Correlation between obesity and overweight and other variables was not significant (Table 1).

## Discussion

The present study, first study on preschool children obesity in Birjand, showed that prevalence of overweight and obesity in children in Birjand kindergartens were 10.6% and 7.6%, respectively. In another study on students in Birjand, the prevalence of overweight and obesity in different age groups was reported as the following:

In 7–12 year-olds 2.2% and 1.2%, in 12–15 year-olds 5.2% and 2.1%, and finally in 15–18 year-olds, 6.1% and 2.1% respectively [8-10].

Comparing the findings of this study with those of three other previous studies showed that overweight and obesity in the 2–5 year-old group has the highest prevalence ratio to the other groups (7–12 and 15–18 year-olds). The increase observed in the prevalence of overweight and obesity in this study compared to other previous ones performed in Birjand can partly be accounted for the type of research community employed. The subjects of the present study (kindergarten children) generally belong to the middle class families which have working mothers, while, the three other studies were conducted on students in primary and high schools in Birjand covering all socio-economic levels such as low, middle, and top.

Another factor that can justify the phenomenon in this study is the time of the research, i.e. 3–5 years later. The prevalence of overweight and obesity over time can grow.

In a study which was carried out on preschool children in Tehran during 2007–2008, prevalence of overweight and obesity in boys were 9.81% and 4.77%, and 10.31% and 4.49% in girls [11]. According to a research on preschool children in Yazd in 2006, overweight and obesity percent was 4.25% and 3.57%, respectively [12]. The prevalence of overweight in the present study is similar to that of Tehrani children, and more than overweight prevalence in children in Yazd. This difference could be due to climate and the children's socio-economical statuses.

Prevalence of childhood overweight and obesity has a growing trend in developed countries. In the USA, 16% of children are overweight and 31% are at the risk of it. Obesity has tripled in this country since 1960 [1].

Based on several studies in different countries, the following results have been reported:

In Greece, 31.9% of 1–5 year-old children were overweight in 2004 [13]. 20.5% of preschool children in Vietnam were overweight and 16.3% were obese in 2007 [14]. 15.2% of Canadian 2–5 year old children were overweight and 6.3% were obese [15]. 16.6% of 2–6 year-old children in Italy were overweight and 8% were obese in 2006 [16]. In Australia, 15% of 4–5 year-old children were overweight and 5% obese in 2004 [17]. 12.3% of 2–5 year-old children in Bahrain were overweight and 8.4% obese in 2009 [18]. In Norway, 13.8% of children were overweight and obese [19]. In China, a study on 2–6 year-old children during 2006 showed that obesity in them was 14.1% and 7.5% in obese and non- obese families, respectively [20]. According to another study in the major cities of China, 18.1% were overweight and obesity was also reported [21]. Studies on preschool Canadian children showed that 25.1% in 1984 and 36% in 1997 were overweight and obese [22]. In India (Amritsar city of Punjab state), 6.42% of 2–5 year-old children were overweight and 2% obese in 2010 [23].

A study in Canada and another study in Australia reported an annual 1% increasing in the prevalence of obesity [24]. Prevalence of obesity in 2–5 year-old children increased from 5.8% to 7.9% in Thailand between 1997 and 2001 [25].

According to a study on preschool children in the East Mediterranean, prevalence of obesity was 3% in the UEA, Iran, and Pakistan and 8.6% in Egypt [26]. This finding is close to ours.

Despite increasing prevalence of overweight and obesity, which is reported from both developed and developing countries, few cases of controlled or decreasing obesity in children in these countries have been reported.

A study in Japan on 5–17 year-old children, between 1997 and 2000 showed that prevalence of overweight and obesity has decreased slowly since early 2000, and the most prevalence has been in the late 1990s [27]. In the Netherlands, prevalence of girls' overweight reduced between 1999 and 2007 [28].

Based on the present study, preschool children's overweight and obesity were significantly correlated with their mothers' jobs and fathers' level of education. This correlation was reported in three other studies two of which were carried out in Birjand, and one Thailand [8-10,25].

The correlation of prevalence of overweight and obesity with parents' education level and occupation can be because of economic and social conditions. These conditions influence behavior, life style, food access facilities, and physical exercises in children and eventually lead to their difference in calorie uptake and its metabolism.

Prevalence of obesity in children is influenced by different factors such as race, environmental and behavioral causes such as socio-economic conditions, urbanization, changes in life style, consumption of high calorie fast foods,and inactivity.

Measures to control and prevent obesity in children are necessary to take,and can be done by life style modification and proper nutrition.

Weight loss and malnutrition have been identified as threats to the health of children in developing countries, and they are conceptions that must be understood correctly.

The limitation of the present study is that in a descriptive study it was not possible to prove any causal relationships between obesity and its risk factors. There is no background on which to compare the present status with the past ones. This is why periodic studies in this field are recommended, especially in this deprived area of Iran.

Another limitation is due to the fact that some 2–4 years old children don^'^t go to kindergarten, so the results of these age groups, may not be indicative of the general population. It is recommended in future studies, children that care in homes are also being studied. But the results of 5 years old,is representive of this age group because of entering of this age groups to kindergarten is mandatory in Iran.

## Conclusion

As a general attitude, overweight and obesity are typical of beauty and health in children. In other words, "a heavy child" means "a healthy child", and the concept "bigger is better" is widely accepted. This false belief leads to a delay in diagnosis, prevention, and treatment of some diseases.

False beliefs are health-threatening, regarding children who are overweight and obese, and must be corrected and families must become aware of the nature of their nutrition. Children must be encouraged to do more exercises from early stages of life. These factors can help us to control children's obesity. Attention to childhood obesity and its measurement is important in community health policies; thus it must be taken into consideration.

Further studies are recommended to identify risk factors in obese children. Periodic studies are necessary to compare the prevalence of obesity of children in future years.

## Abbreviations

TV: Television; BMI: Body Mass Index; CDC: Centers for Disease Control.

## Competing interests

The authors declare that they have no competing interests

## Authors’ contributions

TF and KT designed and oversaw the survey. NA assisted in the planning and co-ordination of the survey. HT MM and SG designed analysis and drafted the manuscript. All authors read and approved the final manuscript.
